# Effect of calcium carbonate particle size and content on the thermal properties of PVC foamed layer used for coated textiles

**DOI:** 10.55730/1300-0527.3514

**Published:** 2022-11-22

**Authors:** Mouna STAMBOULI, Walid CHAOUCH, Sondes GARGOUBI, Riadh ZOUARI, Slah MSAHLI

**Affiliations:** Textile Engineering Laboratory, LGTex, ISET Ksar Hellal, University of Monastir, Monastir, Tunisia

**Keywords:** Synthetic leather, filler, thermal stability

## Abstract

The goal of this research is to see how the amount and particle size of calcium carbonate (CaCO_3_) used in the foamed layer in use for PVC-coated textiles affects the thermal properties of the material. Two different particle sizes were used at various concentrations. The impact of different CaCO_3_ loadings and particle sizes on the PVC foamed layer’s thermal properties were examined. Thermogravimetry (TGA and DTG) and differential scanning calorimetry (DSC) measurements were utilized to investigate the thermal properties of the PVC foamed layer and the samples have been also characterized by FTIR spectroscopy. According to the findings, the thermal stability of the foamed layer was improved with the addition of calcium carbonate. Through the higher surface area between the filler and the PVC matrix, smaller particle sizes have produced the best results. The PVC foamed layer shows also changes in FTIR spectra after adding CaCO_3_, and the intensity of peaks increases with decreasing CaCO_3_ particle size.

## 1. Introduction

Synthetic leather, also called artificial leather or coated textile, is becoming more popular as a substitute for natural leather in a variety of applications thanks to its low cost and its similar look and durability to authentic leather. The coating is increasingly becoming an important way of adding value to textiles. It aims to develop the functional properties of textiles, improve certain characteristics, and guarantee that fabrics meet parameters of performance that will not be achievable from uncoated and leathered fabrics [1].

Polyvinyl chloride (PVC) is one of the most extensively utilized polymers in the coating industry due to its low cost, low density, fire retardancy, excellent insulation, and high mechanical and thermal properties. PVC synthetic leather is widely used in daily life [2–4].

So far, the significance of their properties has been greatly emphasized as a result of their application in various domains such as footwear, automotive, flooring and wall coverings, handbag accessories, medical equipment, and clothing [5,6]. The superficial layer, foamed layer (internal layer), and backing textile are common components of these materials [7].

Their basic components are as follows: PVC resin, a stabilizer, a plasticizer, a blowing agent, and a filler [8]. Plastisol is made by evenly mixing these components [9,10]. Fillers are especially utilized to reduce the cost of the final product. Nevertheless, their properties are a critical factor in defining several technical features of PVC synthetic leather [11–13].

Calcium carbonate is one of the most widely used fillers for the PVC leather industry [14–16], conventionally CaCO_3_ has been employed to minimize its cost and enhance its melting viscosity, and mildly increase the modulus of the final product due to its small surface area and undesirable geometrical aspects [17]. However, some mechanical properties remained constant or, in some circumstances, declined [18]. Particle shape and size and filler quantity have recently been reported to have a significant effect on PVC materials filled with calcium carbonate [19]. Rigorous studies have shown the effect of calcium carbonate on the mechanical behavior of PVC materials [20].

Other studies have shown that CaCO_3_ particle size and content have a significant effect on the morphological structure [17,21,22], the thermal characteristics [16,23,30], and the physical properties of the PVC products [17,21].

However, no published references on the subject of the foamed layer used for PVC synthetic leather have been found.

This study aims to explore the functionality of calcium carbonate in the thermal property enhancement of the PVC foamed layer used for coated textiles and to provide a detailed analysis to demonstrate the impact of CaCO_3_ concentration and particle size on the thermal characteristics of the PVC synthetic leather foamed layer.

## 2. Experimental

### 2.1. Raw materials

PVC resin, Plasticizer (DINP), stabilizer, CaCO_3_ fillers, blowing agent (azodicarbonamide), kicker, transfer paper, pigment, and textile fabric were generously donated by PLASTISS company (Monastir, Tunisia). The different calcium carbonate particle sizes (1.8 μm and 0.9 μm) were provided by SOFAP company (Sfax-Tunisia).

### 2.2. Synthesis of PVC foamed layer

In this study, PVC foamed layers have been developed from PVC plastisol. To make PVC plastisol, 100 parts PVC resin, 80 parts DINP, 4 parts azodicarbonamide, 2 parts Kicker, and 1.5 parts stabilizers were mixed in a mechanical stirrer. Then 25%, 50%, 75%, 100%, or 125% (by weight) of fillers were included and mixed until a consistent mixture was obtained.

The transfer coating technique has been used to create a PVC-foamed layer. The plastisol is applied to the transfer paper with a blade and the thickness is controlled simultaneously. The resultant film, called the foamed layer or internal layer, once it is dried at 200 °C for 80 s and steamed. During plasticizing, the azodicarbonamide (chemical blowing agent) decomposes, generating ammonia gas (NH_3_), which dissipates in the plastisol. Until curing is completed, the gas must remain dissolved in the melting.

### 2.3. Thermal characterization

The TGA and DTG curves of the foamed layers have been analyzed using Perkin Elmer STA 6000 in the temperature range of 0–600 °C at a heating rate of 10 °C min^−1^ under a nitrogen stream and an oxidizing atmosphere.

DSC measurements were carried out at a heating rate of 0.1 °C/min in ambient air conditions, using a Mettler Toledo.

### 2.4. Chemical characterization

The FTIR spectra have been acquired on a Perkin-Elmer BXFTIR system spectrometer (by dispersing samples in KBr disks).

## 3. Results and discussion

### 3.1. FTIR characterization

**Figure 1:**
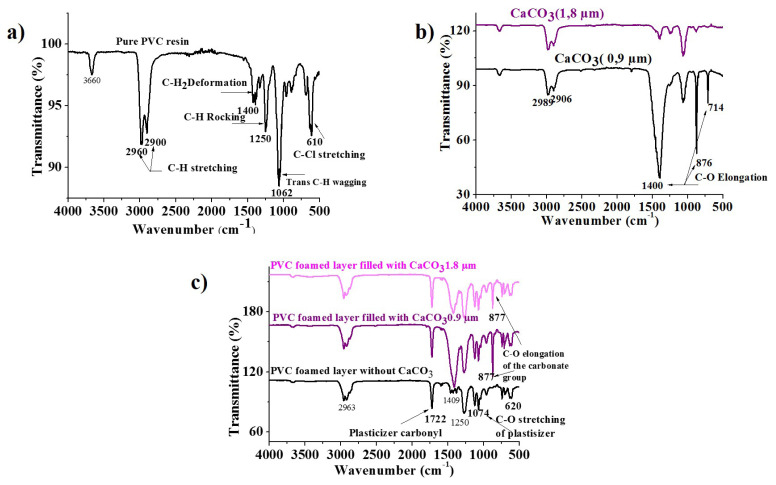
FTIR spectra. a. Pure PVC resin; b. Calcium carbonate with various particle sizes; c. PVC internal layer before and after adding CaCO3 with different particle sizes.

The FTIR spectra of PVC resin, represented in Figure 1a, show the characteristic vibrational modes as summarized in Table 1 [31].

Figure 1b shows the FTIR spectra of calcium carbonate (CaCO_3_), which is characterized by the three C-O elongation modes of the carbonate groups [32]. They appear as a triplet consisting of:

- A large and intense absorption band at 1400 cm^−1^.
- A thin and intense band at 876 cm^−1^.
- A thin and weak band at 714 cm^−1^.

These similar absorption bands were also reported by Wen et al. [33] and Luo et al. [34].

The FTIR spectra of the PVC internal layer with 0% and 50% of CaCO_3_ with different particle sizes (0.9 μm and 1.8 μm) are shown in Figure 1c. The different samples were characterized by different types of elongation modes [35] which appear as several absorption bands and are as follows: the peak set at 2963 cm^−1^ corresponds to the C-H stretch bond [36], the peak located around 1409 cm^−1^ suitable for The C–H aliphatic bending bond, the peak located at 1250 cm^−1^ is attributed to the C-H deformation bond near chlorine (Cl), the peak observed in the 1000–1100 cm^−1^ region corresponds to the PVC backbone chain’s C–C stretch bond. Finally, the peak detected at 620 cm^−1^ represents C–Cl gauche bonds, similar absorption bands have been reported by Atef et al. [37], Lee et al. [38], and Ramesh et al. [36].

The CH_2_ bending peak, with a wavenumber of approximately 1409 cm^−1^, is maximum in the reinforced samples, and the peak intensity increases with decreasing particle size, compared to the FTIR spectra of the reinforced foamed layer with the pure samples as a reference. Another notable difference is the presence of a very thin and sharp peak in the reinforced samples with a wavenumber of around 877 cm^−1^, which can be attributed to the C-O elongation modes of the carbonate groups [39].

### 3.2. Thermal characterization

**Figure 2:**
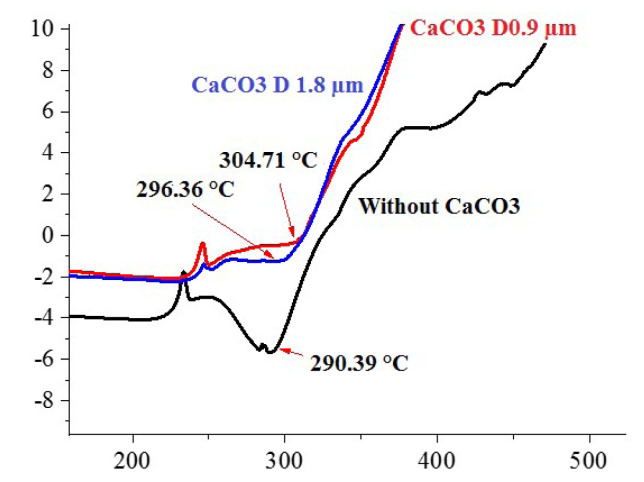
DSC curves of the pure PVC and PVC/50% CaCO_3_ foamed layers prepared with different particle sizes (0.9 μm and 1.8 μm).

Figure 2 shows that the fusion point of the pure PVC foamed layer was detected at 290.39 °C [42]. DSC curves of PVC/50% CaCO_3_ foamed layers with a fine particle diameter (0.9 μm) and PVC/50% CaCO_3_ with a large particle diameter (1.8 μm) have revealed a linked melting peak located around 304.71 °C and 296.36 °C, respectively. It is clear that by adding CaCO_3_, the fusion point of the pure PVC foamed layer was raised to a higher temperature. It can be concluded that calcium carbonate improves the thermal stability of the PVC foamed layer.

Etienne et al. [24], Matthews et al. [27], Tuen et al. [43], Zhu et al. [30], and Sun et al. [28] confirmed these findings and attributed them to the CaCO_3_’s HCl scavenger action during PVC thermal decomposition.

As shown in Figure 2, it has also been demonstrated that thermal stability is improved when using small particle sizes of CaCO_3_ due to their larger surface area, as previously reported by Liu et al. [26]. Calcium carbonate particles with a larger surface area have absorbed the HCl gas released during the PVC thermal decomposition more successfully.

**Figure 3:**
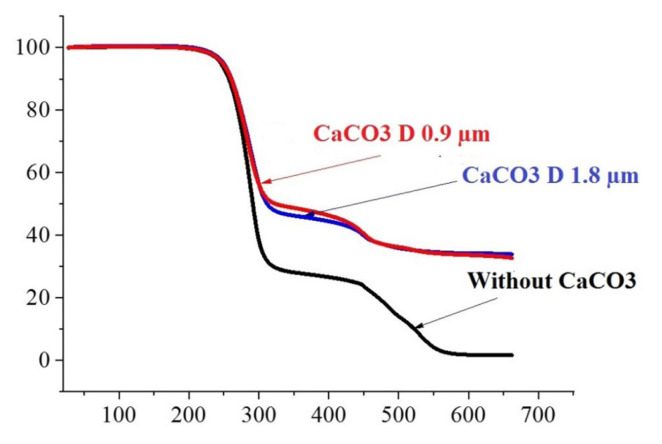
TGA curves of pure PVC and PVC/50% CaCO_3_ foamed layer with different particle sizes (0.9 μm and 1.8 μm).

**Figure 4:**
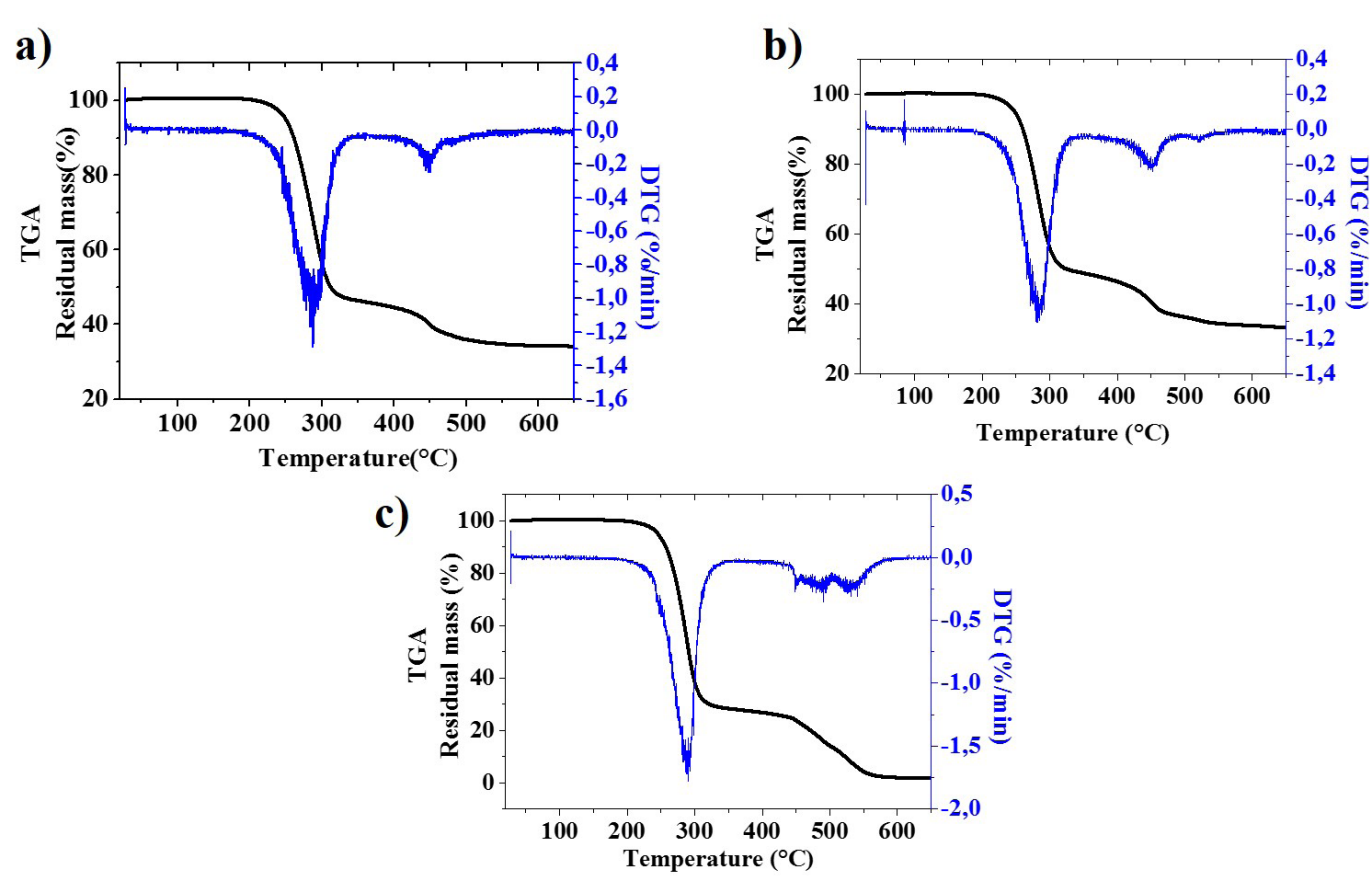
DTG curves. a. PVC/50% CaCO_3_ (0.9 μm); b. PVC/50% CaCO_3_ (1.8 μm); c. pure PVC foamed layer.

Figures 3 and 4 illustrate the TGA weight loss and derivative thermograms (DTG), respectively, for pure PVC and PVC/50% CaCO_3_ foamed layers with varying CaCO_3_ particle sizes. Thermal factors are listed in Table 2.

From the TGA and DTG curves illustrated in Figures 3 and 4, the thermal degradation of pure PVC and PVC/CaCO_3_ foamed layers happens in two main stages, and two substantial weight losses can be seen [24,39].

As also observed by Etienne et al. [24], a minor delay in the two onset decomposition temperatures was noticed (Figure 3) and increased with the use of small CaCO_3_ particle size.

Moreover, the weight loss of the PVC/50% CaCO_3_ foamed layer was lower than the pure PVC foamed layer, and the internal layer filled with small particles typically has the lowest weight loss among the other samples used in this study.

We can conclude that the incorporation of CaCO_3_ can ameliorate the thermal stability of the PVC foamed layer used for PVC synthetic leather, and using a small CaCO_3_ particle size increases the enhancement of the thermal stability. The results of the TGA and DTG analyses were in good agreement with the DSC data.

## 4. Conclusion

The effects of particle size and content of CaCO_3_ filler on the thermal properties of the PVC foamed layer used for synthetic leather were investigated, and we have demonstrated that adding CaCO_3_ can ameliorate the thermal stability of the PVC foamed layer. Therefore, the melting point and the onset decomposition temperatures of the filled foamed layer increase compared with the unfilled one. Many authors attributed these findings to CaCO_3_’s superior ability to trap HCl gas produced during PVC decomposition. Moreover, we observed that the most important positive impact on thermal stability was detected when using a smaller particle size, which has contributed to its larger surface area that can help to consume much more HCl gases, according to several researchers.

## Figures and Tables

**Table 1:** Vibrational modes observed in PVC resin.

Modes of vibration	Wavenumber (cm^−1^)
–CH stretching	2900–2960
–CH_2_ deformation	1400
CH rocking	1250
trans CH wagging	1062
C–Cl stretching	610

**Table 2:** Thermal factors.

Samples	1^st^ decomposition		2^nd^ decomposition	
	T (°C)	Weight loss (%)	T (°C)	Weight loss (%)
Pure PVC	196	72	420	24
PVC/50% CaCO_3_ (1.8 μm)	225	54	421	6.8
PVC/50% CaCO_3_ (0.9 μm)	226	51	423	6.59
